# Quality of Life and Prevalence of Impulsivity in Females With Migraine Headaches

**DOI:** 10.7759/cureus.85649

**Published:** 2025-06-09

**Authors:** Mariwan Husni, Aldana M Zayed, Abdulla AlSheeroqi, Maryam Almusalam, Yousif B Aldoseri, Yusuf Al-Rayyes, Mohamed Elmahdy, Alya Hossam

**Affiliations:** 1 Psychiatry, Northern Ontario School of Medicine (NOSM) University, Thunder Bay, CAN; 2 Pharmacy, Knowledge University, Erbil, IRQ; 3 Psychiatry, King Hamad University Hospital, Busaiteen, BHR; 4 Internal Medicine, Arabian Gulf University, Manama, BHR; 5 Surgery, King Hamad University Hospital, Busaiteen, BHR; 6 Internal Medicine, King Hamad University Hospital, Busaiteen, BHR; 7 Otolaryngology, King Hamad University Hospital, Busaiteen, BHR; 8 Neurology, Bahrain Specialist Hospital, Manama, BHR

**Keywords:** barratt impulsivity scale (bis), borderline personality disorder, depression, migraine disorder, ocd/ anxiety disorders

## Abstract

Background

Migraine is a common neurological disorder with significant socioeconomic and personal impact. Recent research suggests a potential association between migraine and impulsivity, particularly in females.

Objective

This study aims to investigate the prevalence of impulsivity and its correlation with migraine-related disability in females with migraine.

Methods

This is a case-control study involving female patients aged 18-60 with migraine, recruited from neurology and psychiatry outpatient clinics. Standardized assessments, including Migraine Disability Assessment (MIDAS), Patient Health Questionnaire-9 (PHQ-9), Generalized Anxiety Disorder 7-item (GAD-7), Barratt Impulsiveness Scale Version 11 (BIS-11), and McLean Screening Instrument for Borderline Personality Disorder (MSI-BPD), were used to evaluate migraine disability, depression, anxiety, and impulsivity. Data analysis was performed using SPSS version 25 (IBM Corp., Armonk, NY, USA).

Results

There were a total of 149 participants (n=149) in the study, comprising 68 (n=68, 46%) migraine sufferers and 81 (n=81, 54.4%) controls. Migraine patients had significantly higher impulsivity (BIS-11: 34.67 ± 11.95 vs. 26.81 ± 2.99, p < 0.001) and a higher prevalence of borderline personality traits (MSI-BPD: 2.76 ± 2.65 vs. 1.23 ± 0.93, p < 0.01). Depression and anxiety were also significantly more common in the migraine group.

Conclusion

These findings suggest a strong association between migraine, impulsivity, and mood disorders, emphasizing the need for integrated psychological and neurological management in migraine patients.

## Introduction

An estimated 12% of people suffer from migraines. Worldwide estimates are greater. Chronic migraine (CM) affects 1% to 2% of people worldwide. About 2.5% of people who have episodic migraines go on to develop CM [1]. Migraine is a primary headache disorder that has been shown to have a significant impact on individuals' socioeconomic and personal lives [2]. According to the International Headache Society's International Classification of Headache Disorders, third edition (ICHD-3) (2018) [3], migraine is defined as a recurrent episode of moderate to severe headaches, usually unilateral and pulsating. It is a frequent and incapacitating primary headache disorder. In certain cases, aura symptoms may precede or accompany migraines. Other symptoms may include nausea, photophobia, and phonophobia.

Current research suggests that individuals with migraines may be at a higher risk of developing certain personality disorders, which are more common in women [4]. Given the high prevalence and disabling factors of migraine, this study aims to investigate the quality of life and the prevalence of impulsivity in females with migraine headaches.

A study conducted in 2019 analyzed 61 patients with migraine and its comorbidity with personality disorders, revealing that migraine was more severe in those with personality disorders [5,6]. Among personality disorders, Borderline Personality Disorder (BPD) affects 2% of the general population and is more common in females with a defining characteristic of impulsivity [7]. In inpatient settings, 26% of migraine patients have been diagnosed with personality disorders, with 16% presenting with BPD [8]. A clear association has been demonstrated between BPD and chronic, frequent pain, including headaches and migraines [9]. The severity of headaches varies between those with and without personality disorders, with 5.5% experiencing moderate to severe headaches in the presence of BPD [10,11]. Similarly, the prevalence of psychiatric conditions such as personality disorder and impulse control disorder (ICD) in migraine patients is evident, with comorbidity rates ranging from 10 to 10.5% [12]. Previous attempts to demonstrate a connection between migraines and impulsive behavior have not yielded clear results [13]. This study aims to further explore the relationship between migraines and impulsive behavior.

Additionally, non-modifiable and modifiable risk factors, such as gender and smoking, play a role in the prevalence of BPD and ICD. A Swedish study that included around 2 million individuals identified 12,175 cases of BPD, with 85.3% being females [14]. Studies indicate that females have a higher risk of developing BPD, whereas males are more commonly diagnosed with ICD [15]. This study aims to examine the quality of life in females with migraine headaches and the prevalence of impulsive behaviors.

## Materials and methods

This study analyzes the relationship between migraine and the prevalence of personality disorders, with a special interest in impulsivity and borderline personality disorders. The study is a case-control study where patients suffer from headaches and fulfil the diagnostic criteria of migraine according to the ICHD-3 diagnostic criteria [3], which consist of two primary categories of migraine: aura-accompanied and aura-free. At least five episodes of moderate to severe headaches lasting four to seventy-two hours, accompanied by at least two of the following characteristics - unilateral location, pulsating quality, aggravation by routine physical activity, and accompanying symptoms like nausea, vomiting, photophobia, or phonophobia - are considered migraines without aura. At least two episodes of completely reversible aura symptoms, such as visual or sensory abnormalities, should be present in the migraine with aura category. The patient group was compared with a healthy, age-matched control group selected from community-based female volunteers who were approached by the researchers. Volunteers with physical or mental health issues, including headaches, were excluded from the control group.

The inclusion criteria of patients in this study also consist of females aged between 18-60 years of age, who are free from medical illness and do not have an organic cause pertaining to their headache. The patient selection included all the patients who were followed up in the headache outpatient clinic of the neurology department at King Hamad University Hospital (KHUH), Bahrain.

All male patients were excluded from the study. Additionally, any female whose age did not fall within the above-specified range was excluded. Patients with organic causes for headaches, such as intracranial pressure abnormalities, vascular disorders, hypertensive crisis, head and neck disorders, substance use disorders, infections, etc., were not included. Females with any concurrent chronic illness under active management were also excluded. Finally, patients presenting with headaches who were not following up at KHUH, or who were lost for follow-up in the neurology clinic, were excluded from the study.

Written consents were obtained from the patients when distributing the questionnaires in the form of signatures. The method of data collection was subject to approval from the Institutional Review Board of the King Hamad University Hospital research department.

The Migraine Disability Assessment (MIDAS) questionnaire was developed and used to quantify headache-related disability over a three-month period [16], Patient Health Questionnaire-9 (PHQ-9) was used to screen for depression [17], Generalized Anxiety Disorder 7-item scale (GAD-7) was used to screen for anxiety [18], Barratt Impulsiveness Scale Version 11 (BIS-11) was used to measure impulsivity [19], and McLean Screening Instrument for Borderline Personality Disorder (MSI-BPD) was used to screen for borderline personality disorder [20]. The total score for each of the above instruments was calculated and recorded on the Statistical Package for the Social Sciences (SPSS) software.

The data was analyzed using SPSS version 25 (IBM Corp., Armonk, NY, USA). The normality of data distributions was checked with the Kolmogorov-Smirnov test. Chi-square tests were used to compare categorical variables, and t-tests with one-tailed p-value were used to compare the mean score of the measures (PHQ-9, GAD-7, BIS-11 and MSI-BPD), assuming that the patient group would be scoring higher than the control group. One-sample Kolmogorov-Smirnov test (K-S test) was used to evaluate the normality of all variables. PHQ-9 scores and GAD-7 scores have a normal distribution only.

## Results

The sociodemographic characteristics of the patient group, which consisted of 68 individuals diagnosed with migraine headaches, are as follows: The average age of the patients was 36.90 years (±10.11 SD). Most patients were females (91.8%), but only 8.2% were males. In terms of marital status, 29.2% were single, 60.4% were married, and the remaining participants were divorced.

Migraine Disability Assessment (MIDAS) scores assess the impact of migraines on patients' lives. The distribution of MIDAS scores in the patient group was as follows: 26.9% of patients had MIDAS I, indicating minimal disability; 17.9% had MIDAS II, indicating mild disability; 12.0% had MIDAS III, indicating moderate disability; and the most common category was MIDAS IV, observed in 43.3% of patients, indicating severe disability due to migraines.

The Patient Health Questionnaire-9 (PHQ-9) score, which assesses depression severity, had a mean score of 11.41 (±7.02 SD). The distribution of depression severity categories within the patient group was as follows: 17.7% had no depression, 27.9% had mild depression, 26.5% had moderate depression, 13.2% had moderately severe depression, and 14.7% had severe depression.

The Generalized Anxiety Disorder 7-item (GAD-7) score, which measures the severity of anxiety, had an average of 8.51 (± 5.83 SD). The distribution of anxiety severity categories within the patient group was as follows: 25% had no anxiety, 38.2% had mild anxiety, 16.2% had moderate anxiety, and 20.6% had severe anxiety.

The Barratt Impulsiveness Scale-11 (BIS-11) score, which assesses impulsivity, had an average of 34.67 (± 11.95 SD), indicating a relatively high level of impulsivity among the patients.

The McLean Screening Instrument for Borderline Personality Disorder (MSI-BPD) score, which measures the likelihood of borderline personality disorder, revealed an average of 2.76 (± 2.65 SD).

Table 1 below reveals MIDAS, PHQ-9, GAD-7 mean scores and their subgroups, with BIS-11 and MSI-BPD scores for the Patient Group (n=68).

**Table 1:** MIDAS, PHQ-9, GAD-7 mean scores and their subgroups, with BIS-11 and MSI-BPD scores for the Patient Group (n=68).

Migraine Disability Assessment (MIDAS)	33.3 ± 52.22
MIDAS I	18 (26.9%)
MIDAS II	12 (17.9%)
MIDAS III	8 (11.9%)
MIDAS IV	29 (43.3%)
Patient Health Questionnaire-9 (PHQ-9)	11.41 ± 7.02
No depression	12 (17.7%)
Mild Depression	19 (27.9%)
Moderate depression	18 (26.5%)
Moderately severe depression	9 (13.2%)
Severe depression	10 (14.7%)
Generalized Anxiety Disorder 7-item scale (GAD-7)	8.51 ± 5.83
No Anxiety	17 (25%)
Mild Anxiety	26 (38.2%)
Moderate Anxiety	11 (16.2%)
Severe Anxiety	14 (20.6%)
Barratt Impulsiveness Scale Version 11 (BIS-11)	34.67 ± 11.95
McLean Screening Instrument for Borderline Personality Disorder (MSI-BPD)	2.76 ± 2.65

The sociodemographic characteristics of the control group, which consisted of 81 individuals, are as follows: The average age of the control group participants was 34.48 years (±3.71 SD). All participants in the control group were non-smokers. In terms of employment status, 81.5% were employed, and 18.5% were unemployed.

The PHQ-9 score in the control group has a mean score of 5.51 (±1.84 SD). Among the participants, 34.6% exhibited no depression, while 65.4% experienced mild depression. No individuals in the control group fell into the categories of moderate, moderately severe, or severe depression.

The mean GAD-7 score was 7.75 (±3.0 SD), suggesting mild anxiety levels on average. Within the control group, 14.8% reported no anxiety, 55.6% reported mild anxiety, and 29.6% reported moderate anxiety. No severe anxiety was reported.

The control group's impulsivity levels assessed using the Barratt Impulsiveness Scale Version 11 (BIS-11) revealed a mean score of 26.81 (±2.99 SD).

Lastly, the McLean Screening Instrument for Borderline Personality Disorder (MSI-BPD) was employed, confirming a mean score of 1.23 (±0.93 SD).

The above results for the measure in the control group are summarized in Table 2 below.

**Table 2:** PHQ-9, GAD-7 mean scores and their subgroups, with BIS-11 and MSI-BPD scores for the Control Group (n=81)

Patient Health Questionnaire-9 (PHQ-9)	5.51 ± 1.84
No Depression	28 (34.6%)
Mild Depression	53 (65.4%)
Moderate Depression	0
Moderately Severe Depression	0
Severe Depression	0
Generalized Anxiety 7-item (GAD-7)	7.75 ± 3
No Anxiety	12 (14.8%)
Mild Anxiety	45 (55.6%)
Moderate Anxiety	24 (29.6%)
Severe Anxiety	0
Barratt Impulsiveness Scale Version 11 (BIS-11)	26.81 ± 2.99
McLean Screening Instrument for Borderline Personality Disorder (MSI-BPD)	1.23 ± 0.93

**Figure 1:**
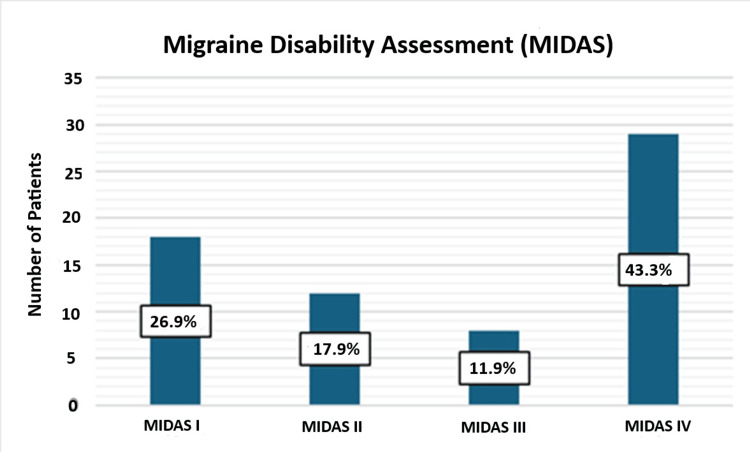
Distribution of Migraine Disability Assessment (MIDAS) scores among the patients

Figure 1 highlights the substantial impact of migraine headaches on the patients' daily lives, as indicated by the significant proportion of patients falling into the moderate and severe disability categories.

The figure visually presents the distribution of MIDAS scores among the patients with migraine headaches. The graph shows the percentage of patients falling into different MIDAS categories. The most common category was MIDAS IV, observed in 29 patients (43.3%), indicating severe disability due to migraines. MIDAS III, indicating moderate disability, was observed in eight patients (12%). MIDAS II, indicating mild disability, which included 12 patients (17.9%), and MIDAS I, indicating minimal disability, was observed in 18 patients (26.9%).

Table 3 below presents the Migraine Disability Assessment (MIDAS) scores for the patient group, as the control group did not have migraine sufferers. It also shows various psychological measures of the patient group and the control group in the study of females with migraine headaches. The patient group exhibited higher MIDAS scores compared to the control group, indicating a greater impact of migraine headaches on their daily functioning. Among the patient group, MIDAS IV was the most common condition, accounting for 43.3% of the patients, suggesting a higher prevalence of chronic migraine. The mean MIDAS score for the patient group was 33.3, with contributions from MIDAS I (26.9%), MIDAS II (17.9%), MIDAS III (11.9%), and MIDAS IV (43.3%). In contrast, the control group did not display any MIDAS scores, indicating the absence of migraine-related disability in this group.

**Table 3:** Compared Measures (MIDAS, PHQ-9, GAD-7, BIS-11 and MSI-BPD) of Patient Group with Control Group

Measure	Patient group (n=68)	Control group (n=81)	t-statistic value	Degrees of freedom (DF)	p-value (1-tailed)
Migraine Disability Assessment (MIDAS)	Mean 33.3 ± 52.22 (SD)	N/A	N/A	N/A	N/A
Patient Health Questionnaire-9 (PHQ-9)	Mean 11.41 ± 7.02 SD	Mean 5.51 ± 1.84 SD	2.57	~10	0.014
Generalized Anxiety 7-item (GAD-7)	Mean 8.51 ± 5.83 SD	7.75 ± 3.00	0.37	~13	0.36
Barratt Impulsiveness Scale Version 11 (BIS-11)	Mean 34.67 ± 11.95 SD	26.81 ± 2.99	2.02	~10	0.034
The McLean Screening Instrument for Borderline Personality Disorder (MSI-BPD)	Mean 2.76 ± 2.65 SD	1.23 ± 0.93	1.72	~10	0.058

The study conducted a comprehensive analysis of sociodemographic characteristics, migraine disability assessment (MIDAS) scores, and psychological measures to examine the differences between the patient group (n=68) and the control group (n=81) of females with migraine headaches. The patient group exhibited a higher average age of 36.90 years (±10.11 SD) compared to the control group's average age of 34.48 years (±3.71 SD). Notably, the patient group consisted predominantly of females (91.8%), while the control group consisted entirely of non-smokers. Marital status analysis revealed that 29.2% of the patients were single, 60.4% were married, and the remaining participants were divorced. Statistical analysis revealed significant differences between the patient and control groups in terms of MIDAS scores (p = 0.000), further supporting the impact of migraine headaches on patients' quality of life.

Table 3 also shows that the patient group scored higher in the Patient Health Questionnaire-9 (PHQ-9), indicating a higher prevalence of depression symptoms among individuals with migraine headaches. The mean PHQ-9 score for the patient group was 11.41 ± 7.02 (SD), with varying levels of depression observed: No Depression (17.7%), Mild Depression (27.9%), Moderate Depression (26.5%), Moderately Severe Depression (13.2%), and Severe Depression (14.7%). In the control group, the mean PHQ-9 score was 5.51 ± 1.84 (SD), with a higher percentage of individuals reporting No Depression (34.6%) and Mild Depression (65.4%). There was also a statistically significant difference in PHQ-9 mean scores between patients and controls, with the t-statistic value of 2.57, degrees of freedom (df) 10, and p-value 0.014.

There were no significant differences in GAD-7 scores (p = 0.36) between the two groups, suggesting that anxiety levels may not differ significantly between those with migraine headaches and the control group (t-statistic value 0.37, degrees of freedom (df) 13 and p-value 0.36). The distribution of anxiety levels in the patient group was as follows: No Anxiety (25%), Mild Anxiety (38.2%), Moderate Anxiety (16.2%), and Severe Anxiety (20.6%). In the control group, the mean GAD-7 score was 7.75 ± 3.0 (SD), with a higher percentage of individuals reporting Mild Anxiety (55.6%) and No Anxiety (14.8%).

Additionally, the patient group displayed higher scores on the Barratt Impulsiveness Scale Version 11 (BIS-11). There was a statistically significant difference of mean scores between the patient and control groups (t-statistic value 2.02, degrees of freedom (df) 10 and p-value 0.034). There was only a borderline significant difference in the mean scores of MSI-BPD between the two groups (t-statistic value 1.72, degrees of freedom (df) 10 and p-value 0.58).

The results of this study emphasize the significant and varied effects of migraines on the daily lives of patients. It is noteworthy that the patient group experienced higher levels of disability, depression, impulsivity, and a moderate likelihood of borderline personality disorder compared to the control group (Tables 1-3 and Figure 1). These findings highlight the urgent requirement for comprehensive strategies to manage and intervene in the physical, emotional, and psychological difficulties faced by women dealing with migraine headaches.

## Discussion

Most patients in this study were females (91.8%), with only 8.2% being males. As the global prevalence of migraines is 20.7% in females and 9.7% in males, this gender disparity refers to hormonal differences as well as differences in brain structure. Also, genetic polymorphisms and neuronal pathways share this disparity [21].

Most migraine patients reported an average of 34.67 (± 11.95 SD) in the BIS-11 score, indicating a relatively high level of impulsivity among the patients. The global prevalence of impulsivity with attention deficit hyperactivity disorder (ADHD) symptoms like impulsiveness is higher in adults with migraines [22]. Impulsivity as isolated symptom is not a part of the ordinary symptomatic course of migraine, but the connection of migraine with certain mood disorders like bipolar disorder and the finding of a high frequency of migraine (44%) among patients with intermittent explosive disorder signify this connection, which suggests that impulsivity and mood fluctuations may be a common link between migraine and ADHD [23]. Both disorders potentially share underlying neurological mechanisms and are affected by genetics. Research based on twin studies, which is a method used to examine the relative contributions of genetics and environment to certain disorders, suggests that Attention-Deficit/Hyperactivity Disorder (ADHD) is highly heritable, with genetic factors accounting for approximately 75% to 90% of the risk. This means that most of the differences in susceptibility to ADHD among individuals can be traced to inherited genetic influences. If one person in a family is diagnosed with ADHD, there is a 25%-35% probability that another family member also has ADHD, compared to a 4%-6% probability for someone in the general population. Similarly, if one parent has migraine disease, there is a 50% chance that their child will inherit it. If two parents have a migraine, there is a 75% chance that their child will also inherit it [22]. In addition, disorganization of habits like hydration and poor eating may play a role in triggering migraines in ADHD patients [24]. On the other hand, as one of the major symptoms of migraine is anticipating chronic pain, this also aids in increasing impulsive behaviors among them. A case control study was conducted to identify aggressiveness in 144 migraine patients and concluded that the overall Autism Spectrum Quotient (AQ) score, and anger and hostility subscale scores were higher in migraine patients than controls, specifically for patients with chronic migraine [25].

In comparison to the control group, the patient group had a higher percentage of moderate depression (26.5%) and anxiety (38.2% mild anxiety, 16.2% moderate anxiety, and 20.6% severe anxiety), and borderline personality disorder had an average of 2.76 (± 2.65 SD), suggesting a moderate likelihood of borderline personality disorder among the patients. This high percentage of co-existing mood disorders refers to the complexity of migraine pathophysiology and the anticipation of episodic or chronic pain that leads to overuse of medications [23]. Many cofactors contribute to the justification for this bidirectional relationship between migraine and mood disorder, including genetics, childhood trauma, hormonal fluctuations, neurotransmitter dysregulation, and impaired regulation of the biology underlying the stress response [26]. A study done in the Neurology Outpatient Department of a Tertiary CARE Hospital from August 1, 2016, to February 28, 2017, examined a total of 133 patients, and it was concluded that the duration and frequency of migraine headaches were found to be positively correlated with the presence of mood disorders. The estimated prevalence of patients with anxiety was 16.54% and with depression was 9.02% in migraineurs [27].

Migraine patients reported a higher disability score - MIDAS score IV was the most common category which indicates severe disability (43.3% of patients) - in comparison to the control group. Migraine is considered the second leading cause of disability in the US and the first among young women in 2019 [28]. In addition, in a systemic review targeting the prevalence of migraine in Saudi Arabia, it was found that The Global Summary Report of the Eastern Mediterranean Region, 1990-2016, stated that Saudi Arabia had the highest rising trend in age-standardized years lived with disability (YLD) rates of migraine, as the estimated pooled proportion of migraine in Saudi Arabia is 0.225617 [29].

Multifactorial higher disability justification may refer to the number of absences from work or school that affect patients’ productivity, socialization, and increased sense of independence, which affects their quality of life in all domains [30]. Long-lasting episodic migraine hinders patients’ ability to concentrate and succeed at work, and migraine sensitivity to light, sound, and noise definitely will affect the socialization of the individuals [30]. As episodic migraine frequency increases, migraine-related productivity loss increases. A study of 5916 workers at a car manufacturing factory in Turkey stated that workers with 10-14 headache days per month had a range of two absences and 46 days with migraine-related impaired productivity per year in comparison to 3.5 days of absences and 87 days with decreased productivity for those with at least 15 days of headache per month who have chronic migraine [31]. In the American Migraine Prevalence and Prevention study, 29% of the 5997 individuals with more than 10 headache days per month accounted for 49% of overall lost productive time.

Patients in this study had higher MSI-BPD scores than controls, although the connection was borderline significant (p=0.058). Prior studies show that Borderline Personality Disorder (BPD) and chronic migraine are significantly comorbid, especially in women. This correlation might result from common characteristics such as hypersensitivity to emotions, hyperreactivity to stress, and autonomic nervous system dysfunction. Furthermore, serotonin and other neurotransmitter system disruptions are frequently present in both disorders. Further clinical examination may reveal a significant relationship, though, as the MSI-BPD is a screening tool rather than a diagnostic one.

This study has certain limitations. Firstly, the study sample size is relatively small. Second, most of the respondents were female, and the collected data was irrespective of their menstruation period. As is known, PMS (pre-menstrual syndrome) is associated with multiple mood swings. These factors may correlate, resulting in unintended biases and outcomes. On the other hand, we have collected data using an in-person questionnaire in a second Bahrain tertiary central hospital. Our study was based on standardized measurements to categorize degrees of depression, anxiety, and impulsiveness in migraine patients, which increased the validity and reliability of the outcomes.

Further studies with a larger sample targeting different demographics are needed for a better understanding of the different factors influencing correlation outcomes and the bidirectional relationship between migraine disorder and mood and personality disorders, which may lead to more effective interventions. 

Clinicians should focus routinely on migraine disability level and quality of life in patients, as it needs a complementary approach and a multidisciplinary team to ensure that patients are receiving proper treatment and whether any additional strategies are needed or not. A detailed history and diagnostic approaches should take into consideration the bidirectional relationship and synergistic effects between migraine disorder and mood and personality disorders to determine optimal interventions.

## Conclusions

Migraine is common in females, who have a high level of impulsivity in migraine. Coexistence of depression and anxiety among migraine sufferers underscores the intricate nature of migraines and the impact of chronic pain on mental well-being. The frequency and duration of migraine are correlated with the presence of mood disorders.

Migraine causes a substantial disability, adversely impacting productivity and quality of life. This study emphasizes the importance of addressing these comorbidities to enhance patient outcomes. Clinicians need to adopt a comprehensive and holistic approach to improve the quality of life for migraine patients. Effective management of migraine should include tackling associated mood and personality disorders.
